# The principle of equivalence as a criterion of identity

**DOI:** 10.1007/s11229-018-01897-w

**Published:** 2018-10-04

**Authors:** Ryan Samaroo

**Affiliations:** 1 Department of Philosophy, University of Bristol, Cotham House, Bristol, BS6 6JL UK; 2 Somerville College, University of Oxford, Woodstock Road, Oxford, OX2 6HD UK

**Keywords:** Principle of equivalence, Criterion of identity, Foundations of space-time theories, Frege, Einstein, Newton

## Abstract

In 1907 Einstein had the insight that bodies in free fall do not “feel” their own weight. This has been formalized in what is called “the principle of equivalence.” The principle motivated a critical analysis of the Newtonian and special-relativistic concepts of inertia, and it was indispensable to Einstein’s development of his theory of gravitation. A great deal has been written about the principle. Nearly all of this work has focused on the *content* of the principle and whether it has any content in Einsteinian gravitation, but more remains to be said about its *methodological role* in the development of the theory. I argue that the principle should be understood as a kind of foundational principle known as a *criterion of identity*. This work extends and substantiates a recent account of the notion of a criterion of identity by William Demopoulos. Demopoulos argues that the notion can be employed more widely than in the foundations of arithmetic and that we see this in the development of physical theories, in particular space–time theories. This new account forms the basis of a general framework for applying a number of mathematical theories and for distinguishing between applied mathematical theories that are and are not empirically constrained.

## Introduction

“The principle of equivalence,” which Einstein originally used to refer to one particular statement, has come to refer to a number of interrelated principles in the theory of gravitation.^1^ The principle formalizes an insight into gravitation that Einstein had in 1907. This is the insight, roughly speaking, that objects in free fall do not “feel” their own weight. The principle motivated a critical analysis of the Newtonian and 1905 inertial frame concepts, and it was indispensable to Einstein’s argument for a new concept of inertial motion.

But setting aside its role in Einstein’s argument, the principle is generally held to be one of the foundations of Einstein’s theory of gravitation, even if the sense in which it is a foundation is disputed. For these reasons, a great deal has been written about the principle. Nearly all of this work has focused on the *content* of the principle and whether indeed it has any content in Einsteinian gravitation, but more remains to be said about its *methodological role* in the development of the theory. A methodological analysis must consider two basic questions: what kind of principle is the equivalence principle? What is its role in the conceptual framework of gravitation theory? I maintain that the existing answers are unsatisfactory and I offer new answers. I argue that the equivalence principle should be understood as expressing a kind of foundational principle known as a *criterion of identity*. The principle functions as a criterion for recognizing when the motions of different reference frames are the same motion; it has the consequence that the motion of an inertial frame is the same as the motion of locally freely falling frame. My new account illuminates the methodological role of the equivalence principle in the conceptual framework of gravitation theory and also our understanding of the application of the theory of pseudo-Riemannian manifolds in Einsteinian gravitation. Furthermore, this account of the role of the principle informs our understanding of Einstein’s analysis of the inertial frame concept, and so of the transition from the conceptual framework of special relativity to that of the general theory.

This is a novel use of the notion of a criterion of identity, one that may be surprising even to those already acquainted with the literature in the philosophy of mathematics and the metaphysics of individuals. This study owes several things to the former and nothing to the latter. It builds on the recent account of the notion of a criterion of identity by Demopoulos in *Logicism and its Philosophical Legacy* (2013). Demopoulos argues that the notion of a criterion of identity can be employed more widely than in the foundations of arithmetic and that we can see this in the development of physical theories, in particular space–time theories. Demopoulos’ contribution is a penetrating analysis of the criterion of identity and this study aims to further extend and substantiate it. Although this employment of the notion of a criterion of identity in the foundations of space–time theories may seem at first surprising and even questionable, I hope to show that it is in fact a natural one and that in the case of the equivalence principle it allows us to recover the features of the gravitational interaction that the principle is generally held to establish.

In Sect. 2 I will introduce the 1905 inertial frame concept. Since the equivalence principle motivates a critical analysis of this concept, I will present the concept in overview, beginning with its Newtonian and nineteenth-century antecedents. In Sect. 3 I will consider a number of principles that have been called “the equivalence principle,” and I will examine the relations between them. I will draw attention to one particular principle that, I will argue, fully captures Einstein’s insight of 1907. In Sect. 4 I will survey and evaluate some notable accounts of the principle. In Sect. 5 I will present Demopoulos’ account of the criterion of identity and his claim that other criteria of identity underlie the analysis and interpretation of a number of space–time theories. I will argue that understanding the equivalence principle as a criterion of identity illuminates its methodological role in the conceptual framework of gravitation theory. Last, in Sect. 6, I will examine a few implications of this account. I will show, in particular, that it isolates what is distinctive about Einstein’s contribution to our understanding of the gravitational interaction.

## Background: the 1905 inertial frame

Let us begin by getting clear on the concept of an inertial frame, specifically, Einstein’s 1905 concept that was the object of his 1907 analysis. It is instructive to introduce this concept by way of Newton’s.

Newton’s laws of motion express empirical criteria for the application of the basic concepts of mechanics, namely force, mass, and inertial motion—and all those concepts that depend on them. Inertial motion is that state in which a body moves in uniform rectilinear motion unless acted upon by a force.^2^ Now associate with a body moving inertially a reference frame. In the most general sense, a frame is a space. It is a “small” space in the sense that it is sufficiently local, homogeneous, and isotropic; furthermore, it is a space in which an accelerometer would detect no acceleration. We can give a geometrical description of bodies in the space among themselves using a coordinate system. But we can do more than just give a geometrical description: in any such space, the outcomes of mechanical experiments, calculated using the laws of motion, will be the same. (And the same outcomes would be calculated in any space in uniform rectilinear motion relative to it—this is the Galilei-Newton relativity principle.) This is the Newtonian concept of an inertial frame.

While these frames are empirically indistinguishable, for Newton, they were not theoretically equivalent: Newton thought of them as moving with various velocities relative to what he called “absolute space,” even though those velocities cannot be known. Although Newton introduced this term to refer to the resting backdrop against which we can talk about uniformly moving relative spaces, many of his contemporaries understood it to have certain ontological implications and criticized its introduction on those grounds. It was not until the nineteenth century that Newton’s theory was given its proper form, by the insight into its complete independence from the notion of absolute space, in the work of Neumann (1870), Thomson (1884), Lange (1885), and others.^3^ The nineteenth-century inertial frame concept was the outcome of their work. This is the concept that is assumed at the start of Sect. 1 in the 1905 paper and that Einstein subjects to a critical analysis.

In “On the Electrodynamics of Moving Bodies” (1905), Einstein showed that the Newtonian framework uncritically assumes that two inertial frames agree on whether spatially separated events happen simultaneously. He showed that determining whether two spatially separated events are simultaneous depends on a process of signalling. Beginning with the empirical hypothesis that the velocity of light is the same in all reference frames, Einstein showed that a criterion involving emitted and reflected light signals allows us to judge when two spatially separated events occur simultaneously. This criterion—the *Einstein synchronization criterion*—is expressed as follows: given two locations A and B, and having placed a clock at each, an event at A occurs at the same time as an event at B when “the ‘time’ required by light to travel from A to B equals the ‘time’ it requires to travel from B to A” (Einstein 1905 [1952], p. 40). With this criterion, Einstein showed that inertial frames can agree on the invariance of the velocity of light only if they disagree on which events are simultaneous, and he showed that from this criterion the Lorentz transformations can be derived.

With Einstein’s analysis of simultaneity, no longer could the laws of motion be taken as the sole empirical criteria for constructing an inertial frame. A new criterion—one based on the hypothesis that the velocity of light is the same in all reference frames—is needed. In this way the nineteenth-century inertial frame concept was replaced by the 1905 inertial frame concept: an inertial frame is not merely one in uniform rectilinear motion in which the outcomes of all mechanical experiments are the same but one, furthermore, in which light travels equal distances in equal times in arbitrary directions and in which the outcomes of electrodynamical experiments are the same.^4^ (These frames are related not by the Galilean transformations but by the Lorentz transformations, and a number of quantities that were invariant under the Galilean transformations are revealed to be frame-relative.) This is the 1905 inertial frame or “Lorentz frame,” and it was *this* concept that Einstein subjected to a critical analysis in 1907. That analysis turns on an insight into gravitation that has been formalized in what is called “the equivalence principle.”

## The equivalence principle

“The equivalence principle” has come to refer to a great many principles, all of which capture one or another feature of gravitation. To some, the fact that there are so many versions suggests that the equivalence principle has at best a chequered status. I contend that this is not a problem. That there are many versions should neither surprise nor concern us, though it is reason for taking care to identify the particular features of gravitation that they isolate.

In what follows, I will offer a brief account of the work that led to the principle that encapsulates Einstein’s insight of 1907. There are a number of ways one might organize such an account. For present purposes, it is important to draw a distinction between those versions of the principle formulated in the *context of theory development* and those formulated in the *context of Einstein’s completed gravitation theory*. For now, our focus will be solely on the former; we will turn to the latter further on.

By “the equivalence principle,” some will think immediately, and with some reason, of the *principle of the universality of free fall*, also known as Galileo’s principle and the principle of the uniqueness of free fall. This is the assertion that all bodies fall with the same acceleration in the same gravitational field. It may also be stated as follows: the path of a body in a given gravitational field is independent of its mass and composition. This is the principle that Galileo confirmed to a high degree of accuracy with experiments involving a pendulum and an inclined plane; Newton also tested it by way of pendulum experiments.

Another statement with the same empirical content can be found in Newtonian gravitation. As is well known, Newton’s theory contains two different concepts of mass: inertial mass *m*, the quantity that figures in the second law, that is, the measure of a body’s resistance to acceleration; gravitational mass *µ*, the quantity that figures in the inverse-square law and that is the gravitational analogue of electric charge. It is well-established experimentally that the ratio of gravitational mass to inertial mass is the same for all bodies to a high degree of accuracy. And once we accept that the ratio is a constant, we can choose to use units of measurement that make the two masses for any body equal, so that *µ*/*m* = 1. In this way we can ignore the distinction between gravitational mass and inertial mass. This is summarized in what is often called the *weak equivalence principle*: inertial mass is equivalent or proportional to gravitational mass.^5^ This statement implies that the acceleration of any body due to a gravitational field is independent of its mass and composition.^6^

The term “equivalence principle,” however, is generally associated with the statements that are intended to capture Einstein’s insight into gravitation in 1907. This is the insight, roughly speaking, that bodies in free fall do not “feel” their own weight. I will not state the principle directly, but by way of the interpretive extrapolation from the universality of free fall—the thought experiment—that Einstein himself used to motivate the principle: this is what we now know as “Einstein’s elevator.”^7^ Introducing the principle in this way is instructive; it will also fix a few ideas that will be helpful later on.

There are two versions of Einstein’s elevator: the “gravity-producing” version and the “transforming-away” version. Consider the gravity-producing version. Suppose you stand in an elevator cabin from which you cannot see out. You feel a “gravitational force” towards the floor, just as you would at home. But you have no way of excluding the possibility that the cabin is part of a rocket moving with acceleration *g* in free space and that the force you feel is an accelerative force. Particles dropped in the cabin will fall with the same acceleration regardless of their mass or composition. Einstein also runs the thought experiment the other way: you are inside the elevator cabin. You feel no gravitational force, just as in free space. But you have no way of excluding the possibility that the cabin is falling freely in a gravitational field.

Although Einstein considers both versions, he held the latter to be problematic: true gravitational fields are never transformed away or cancelled by free fall; furthermore, what is transformed away in the thought experiment is only the homogeneous gravitational field. In practice, there *is* a way of distinguishing locally between a freely falling cabin and a cabin in free space. For example, an astronaut in a space shuttle that is freely falling in the gravitational field of the Earth could perform experiments to determine that a water droplet is not spherical but prolate, that is, to determine that it is subject to a “tidal effect” and lengthened towards the source of the field.^8^ For this reason, Einstein attached particular importance to the gravity-producing version and formalized its empirical content in the statement that we might call *Einstein’s own equivalence principle*: it is impossible to distinguish locally between immersion in a homogeneous gravitational field and uniform acceleration.^9^ The field produced by a uniform acceleration is not a mere “inertial field”; it is not simulated or pseudo-gravity, but a genuine homogeneous gravitational field.

But in spite of Einstein’s stating the equivalence principle in this particular way, for the reasons just given, the transforming-away version of Einstein’s elevator and the corresponding principle is essential to the 1907 insight and ultimately more important. We might state the corresponding equivalence principle as follows: so far as tidal effects can be ignored, the outcome of any mechanical experiment performed in a freely falling frame is the same as would be obtained in a Lorentz frame. It was by way of the transforming-away version that Einstein began to recognize that inertially moving frames and freely falling frames are different presentations of the same motion.

But this is not the full extent of Einstein’s insight of 1907. Einstein argued from the principle that all bodies fall with the same acceleration in the same gravitational field to a stronger hypothesis: not only the outcome of any mechanical experiment but that of any non-gravitational experiment performed in a freely falling frame is the same as would be obtained in a Lorentz frame.^10^ Einstein’s bold hypothesis is summarized in the *principle of universal coupling*: all physical processes couple to gravitation and couple in the same way.^11^

This hypothesis was motivated by Einstein’s conviction that there are no physical phenomena that are unaffected by gravitation, that couple differently to gravitation, and hence that could distinguish a freely falling frame from a Lorentz frame. Einstein expressed this as follows:

If there were to exist just one single thing that falls in the gravitational field differently from all the other things, then with its help the observer could recognize that he is in a gravitational field and is falling in it. If such a thing does not exist, however—as experience has shown with great precision—then the observer lacks any objective ground on which to consider himself as falling in a gravitational field. Rather, he has the right to consider his state as one of rest and his surroundings as field-free with respect to gravitation. (Einstein 1920, pp. 265–266; trans. DiSalle 2006)

Without the assumption of universal coupling, something that falls differently from all other things would be a basis for measuring the acceleration of a freely falling particle. Suppose, for example, that electromagnetic phenomena did not couple to gravitation in the same way as Newtonian particles; then the acceleration of a freely falling particle could be measured relative to electromagnetically accelerated trajectories. The assumption of universal coupling implies that, if any phenomena failed to couple to gravitation or coupled in a different way, they would indicate the existence of a background-structure distinguishable from the gravitational field. As Will has put it, the assumption allows us to “discuss the metric **g** as a property of space–time itself rather than as a field over space–time” (Will 1993, p. 68).

The transforming-away version of Einstein’s own equivalence principle, together with the principle of universal coupling, leads us to another version. I will refer to it simply as the *equivalence principle*^12^:

So far as tidal effects can be ignored, the outcome of any local non-gravitational experiment performed in a freely falling frame is the same as would be obtained in a Lorentz frame.

This formulation emphasizes that, at least in a sufficiently local region of space–time, no test can distinguish freely falling frames from Lorentz frames.^13^*This* is the principle that fully captures Einstein’s insight of 1907. Hereafter, when I write “the equivalence principle,” it is to this principle that I am referring.

What does this principle establish? It establishes that the Lorentz frame is not *uniquely* determined by its empirical criteria.^14^ For this conclusion to hold, it is important to acknowledge an idealization that we have been making explicitly all along: this is the qualification, “so far as tidal forces can be ignored.” Ohanian (1977, Section III) perhaps better than anyone else has stressed that there *are* experiments that are sensitive to tidal effects. But he stresses in the same measure that, so far as we acknowledge the idealization and restrict attention to those experiments that are not sensitive to tidal forces, we gain an important insight into gravitation, namely that a locally freely falling frame cannot be distinguished from a Lorentz frame. Or, to put this yet another way, the equivalence principle expresses the conditions under which we can judge that the motions of freely falling frames and Lorentz frames are identical. The principle leads us to recognize these motions as *different presentations* of the *same motion*.^15^

It is worth noting that the equivalence principle has both destructive and constructive aspects. The principle is destructive because it fatally undermines the uniqueness of the Lorentz frame. That is to say, the principle establishes that the Lorentz frame is not uniquely determined by its empirical criteria. It is constructive because it motivates a new inertial frame concept—a new concept of “natural motion.”

## What kind of principle is the equivalence principle?

With a clear understanding of the content of the equivalence principle—at least in the context of theory development—let us turn our attention to its role. A great deal has already been written about the principle, by physicists, historians of physics, and philosophers of physics.

Physicists have examined the evidential basis for the principle (e.g., Dicke 1964; Will 1993), the principle’s approximate character and the scope of its applicability (e.g. Pauli 1921 [1958]; Eddington 1923; Synge 1960; Ohanian 1977), and the problem of expressing the content of the principle in Einsteinian gravitation (e.g., Trautman 1966; Anderson 1967; Anderson and Gautreau 1969). Others, among them historians of physics, have focused on Einstein’s understanding of the principle (e.g., Pais 1982; Norton 1985). Philosophers of physics have examined a range of issues: the significance of particular statements of the principle (e.g., Ghins and Budden 2001); the question of its methodological character (e.g., Friedman 2001, 2010); the significance of the principle for the correct mathematical setting of Newtonian gravitation (e.g., Knox 2014); the question whether quantum mechanics poses a challenge to the principle (e.g., Okon and Callendar 2011).

Virtually all of this work has focused on the *content* of the principle and whether indeed it has any content in Einsteinian gravitation. But there is more to say about its *methodological role* in the context of theory development. A methodological analysis asks the following questions: what kind of principle is the equivalence principle? What is its role in the conceptual framework of gravitation theory? Furthermore, those answers that have been given tend to reflect deflationist and what might be called “eliminativist” accounts of the principle. An example of the deflationist account can be found in the preface to Synge’s *Relativity: The General Theory*:

I have never been able to understand this principle […] The principle performed the essential office of midwife at the birth of general relativity […] I suggest that the midwife be now buried with appropriate honours. (Synge 1960, pp. ix–x)

The suggestion in this and other remarks is that the principle is a mere heuristic, a ladder to be kicked away. Its value lies, or so it is often put, in the fact that it motivates differential equations of a certain form. The principle is no doubt a heuristic, in the strict sense of the word: a search principle. It is part of the empirical basis, the “physical strategy,” that motivated Einstein’s development of his gravitation theory. But the idea that the principle is a “mere” heuristic is problematic. For one thing, the fact that it does motivate certain equations, that those equations are adequate to the description and prediction of physical phenomena, and that a violation of the principle would result in different equations undermines the idea that the principle is a mere heuristic.

The aspiring eliminativist is motivated by a counterfactual: if Einstein had not developed his gravitation theory in 1915, particle physicists would have 20 years later and without the help of the equivalence principle. The eliminativist account, which is suggested in the work of a number of twentieth-century figures and which is implicit in the recent work of Pitts (2016a, b, 2018), is fleshed out with the help of the massless spin-2, and to a lesser extent massive spin-0 and spin-2, theories of gravity.^16^ These research programs assume the framework of relativistic field theory and a “graviton” field, and from these and other assumptions recover versions and relatives, both close and distant, of Einstein’s field equations. In the massless spin-2 theory, the equivalence principle holds and it becomes a theorem, a consequence or feature of the field equations, rather than a foundational principle; in the massive theories, the principle is violated.^17^

There are three main objections to the eliminativist account. First, of these alternative theories, only the massless spin-2 one recovers precisely Einstein’s equations, and then only in their source-free linearized form. The massive spin-0 theory—empirically refuted since 1919 since it does not “bend light”—gives a single equation that is not part of, or compatible with, Einstein’s equations; it is not intended as a modern competitor in its own right. The massive spin-2 equations are all different from Einstein’s equations, albeit subtly. Second, the equivalence principle is, so far as we know, exceptionless; therefore, the theories in which it is violated must, at a minimum, bring something to our understanding of gravitation that outweighs the cost. Third, although it is true that there are multiple paths to a subset of Einstein’s equations, there is a feature of gravitation—the identity of freely falling frames and Lorentz frames—that the principle singles out. This feature is integral to our understanding of gravitation and the principle not only singles it out but ties it to a number of other concepts. For these reasons, the alternative theories of gravity can hardly be said to support a successful eliminativist account since none of them allow us to recover the full Einstein field equations, which are founded on the principle.

A notable exception to the deflationist and eliminativist accounts is that offered by Michael Friedman (e.g., 2001, 2010), who offers an account of the principle’s methodological role. Friedman claims that the equivalence principle is a “constitutive principle”:

[The theory of pseudo-Riemannian manifolds and the equivalence principle] function rather as necessary parts of the language or conceptual framework within which [the field equations make] both mathematical and empirical sense. (Friedman 2001, p. 39)

This is, roughly speaking, the claim that the equivalence principle is a condition of the possibility of conceiving of gravitation as a geometrical phenomenon. I have argued (Samaroo 2015) against Friedman’s account of the principle’s methodological role, though I maintain that something close to Friedman’s view is defensible.

What, then, is the correct account of the equivalence principle’s methodological role? In what follows I develop a new account that draws on and extends Demopoulos’ recent work.

## The criterion of identity

The notion of a criterion of identity has come to be bound up with a number of philosophical programs and a large literature, but the sense at issue here is that found in the *Foundations of Arithmetic* (1884 [1989]). I will present Frege’s use of the notion of a criterion of identity in his analysis of number. Then I will consider Demopoulos’ account of the criterion of identity and I will show that it provides the basis for a new account of the equivalence principle’s methodological role.

### For numbers

The notion of a criterion of identity has its origin in Frege, who made it the cornerstone of his theory of number.^18^ Frege sought to provide an analysis of the concept of number by showing that the theory of the natural numbers can be derived from a principle that has the same scope and generality as conceptual thought itself. The principle in question is the following: for any concepts *F* and *G*, the number of *F*s is the same as the number of *G*s if, and only if, there is a one-to-one correspondence between the *F*s and the *G*s. (Frege 1884 [1989], section 62–63) Frege introduced this principle as a criterion for assessing the conditions under which we should judge that the same number has been presented to us in two different ways, as the number of two different concepts.

In the context of second-order logic, this principle—Frege’s *criterion of identity for numbers*—implies the Peano-Dedekind axioms. But its role does not end there: the criterion also underlies our judgements of equinumerosity in our *applications* of the theory of the natural numbers, for example, our everyday application of the theory of the natural numbers in counting. In this way, the criterion of identity controls the application of the theory of the natural numbers.

Let me elaborate on the sense in which the criterion “controls the application” of the theory. To apply arithmetic, we need to explain the notion of the *number of a concept*, that is, the content of an ascription of number involves the predication of something of a concept. For example, the concept *horse that draws the King’s carriage* has the property of having four objects falling under it. For Frege, this notion is explained when we have a means of judging that two concepts have the same number of objects falling under them—a judgement established by our grasp of the equivalence relation (equinumerosity) that is appealed to in the criterion of identity. Hence, the criterion of identity “controls the application” of arithmetic in the sense that it is a necessary presupposition of our ability to make judgements of equinumerosity, and so of our ability to count.

The question of the methodological character of the criterion of identity is the subject of enduring interest and dispute. To some, taking the criterion as the basis for the construction of the natural numbers is questionable since it seems to presuppose the notion of number, and so makes the construction circular. Because of this supposed circularity, it has been argued, notably by Wright (1997), that the principle is an “abstraction principle,” a stipulation governing the use of a new class of terms. But Demopoulos has worked to restore a proper understanding of the criterion as the expression of an *analysis* of a preexisting notion: number. The criterion captures explicitly what our pre-analytic notion of numerical identity implicitly presupposes; it is this notion that is at work in our applied arithmetical reasoning.

Setting aside this work on its methodological character, Demopoulos claims that the notion of a criterion of identity has a role to play in the analysis of physics. Let us now turn our attention to his argument in *Logicism and its Philosophical Legacy* (2013).

### For lengths

*Logicism and its Philosophical Legacy* (2013) brings together a number of contributions to the foundations of mathematics and physics, the philosophy of science, and philosophy in general. But there is a line of argument that is particularly relevant to this study: the notion of a criterion of identity can be employed more widely than in the foundations of arithmetic and we see this in the development of physical theories, in particular space–time theories.^19^ Demopoulos claims, furthermore, that Frege’s notion of a criterion of identity can form the basis for a new account of the *application* of a number of mathematical theories: just as Frege showed that his criterion of identity for numbers controls the application of the theory of the natural numbers in counting, other criteria of identity control the application of other mathematical theories, among them, Euclidean and Minkowskian geometry. Moreover, Demopoulos claims that the criterion of identity forms the basis of a general framework for distinguishing between applied mathematical theories that are and are not empirically constrained.

To defend these claims, Demopoulos examines Einstein’s account of the application of geometrical theories in “Geometry and Experience” (1921 [1922]). In this Einstein gave an analysis of the concept of length—and through this, an analysis of the basic equivalence relation (congruence) of Euclidean geometry. He points out that congruence can be understood only by way of the principle of free mobility, which interprets the concept by expressing a criterion for its application. The *principle of free mobility* is as follows: if two tracts are found to be congruent once and anywhere, they are congruent always and everywhere. (Einstein used the term “tract,” which is the translation of the German *Strecke,* to refer to a bounded line-segment on a practically rigid body.) The principle is a presupposition of our ability to make measurements of length using a measuring rod, a chain, or a pair of dividers; it is a presupposition of our ability to carry out the compass-and-straightedge constructions of classical geometry, on homogeneous spaces.^20^ In this way the principle controls the application of Euclidean geometry.^21^

Demopoulos argues that the principle of free mobility functions as a *criterion of identity for the lengths of tracts*: the length of one tract is the same as the length of another if, and only if, the tracts are congruent. (Demopoulos 2013, p. 39) The criterion expresses the conditions under which we can judge that the lengths of tracts are identical. In the same way that Frege’s criterion of identity is an analysis of equinumerosity and number, this criterion of identity is an analysis of congruence and length. The criterion makes explicit our pre-analytic conception of the free mobility of practically rigid bodies: the conception that is manifested in our capacity for making ordinary judgements of size, shape, spatial orientation, and distance; the conception that is the basis for geometric constructions, and so for constructive proofs.^22^

Like the criterion of identity for numbers, this criterion of identity also controls the application of a mathematical theory, in this case, Euclidean geometry. But there is an important difference between these criteria of identity: the criterion of identity for the lengths of tracts is *empirically constrained*. Because it is empirically constrained, applied Euclidean geometry is transformed into a part of empirical science: it becomes a part of physics. It is just this difference that secures the apriority of arithmetic, the case for which Frege argued.

It is one of Demopoulos’ principal goals to clarify the relative epistemic status of applied arithmetic and applied geometry—to show why the different epistemic status of arithmetic and geometry has nothing to do with one being “pure” and the other “applied.” Both the Peano-Dedekind theory of the natural numbers and Euclidean geometry can be applied, but the application of the latter transforms it into a part of physics, while in applications arithmetic retains its status as a mathematical theory. It is this point that, before Demopoulos’ analysis, had not been properly made. As we will see, Demopoulos’ employment of the notion of a criterion of identity to clarify both the application of mathematical theories and the distinction between applied mathematical theories that are and are not empirically constrained is vindicated by considering two further cases.

### For times

Demopoulos then turns to Einstein’s analysis of time. In “On the Electrodynamics of Moving Bodies” (1905) Einstein gave an analysis of time—and through this, an analysis of the basic equivalence relation (relative simultaneity) of what would later be called “Minkowskian geometry.” This analysis was offered as a solution to a problem at once practical and theoretical: the problem of determining when spatially separated events occur at the same time. Einstein showed that this problem can be solved by a procedure involving emitted and reflected light signals, as outlined in Sect. 1.

Demopoulos offers a close reading of Einstein’s 1905 analysis of time. He shows that this analysis turns on Einstein’s provision of a criterion of identity for the times of occurrence of spatially separated events. To illuminate Einstein’s analysis, Demopoulos uses a now-familiar geometrical construction. Consider the world-line of some inertial observer *O*. Choose point-events *e*_*1*_ and *e*_*2*_ on *O*. Construct a forward light cone emanating from *e*_*1*_ and a backward light cone emanating from *e*_*2*_ such that they swallow one another. *e*_*1*_ and *e*_*2*_, together with the two new points (label one of them *q*), are the vertices of a “light parallelogram.” Now construct a line through the newly-created vertices; label it *Sim*_*O*_. Label the point of intersection of *O* and *Sim*_*O*_*p*. *Sim*_*O*_ represents the plane of simultaneity relative to *O* and it is the set of all events that are simultaneous with *p*. (Note that we can just as well take another inertial world-line as a starting point and construct its corresponding plane of simultaneity in the same way).
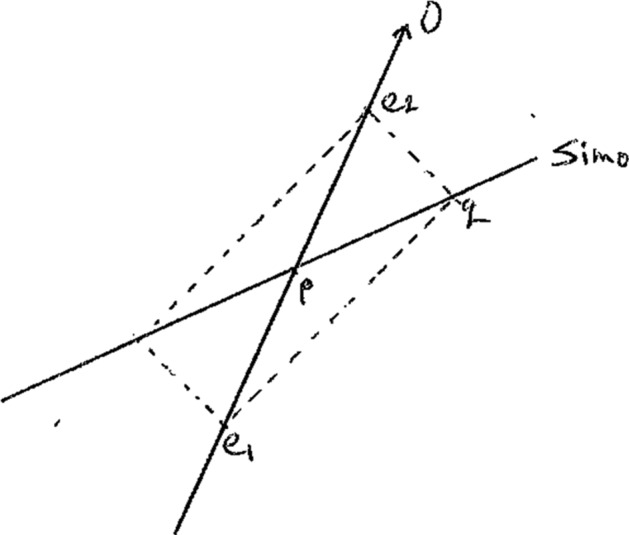


This construction incorporates the Einstein synchronization criterion: it lies in *Sim*_*O*_’s bisection of the interval between *e*_*1*_ and *e*_*2*_.

Demopoulos (2013, pp. 37–38) argues that the synchronization criterion functions as a *criterion of identity for the times of occurrence of spatially separated events*: the time of occurrence of a distant event *q* is the same as the time of occurrence of *p* if, and only if, *p* bisects the interval along *O* that is bounded by the events marking the emission and reception of a light signal sent from *e*_*1*_ and reflected back from a distant event *q*. The criterion expresses the conditions under which we can judge that the times of occurrence of *p* and *q* are identical.

As in the case of the criterion of identity for the lengths of tracts, this criterion of identity controls the application of a mathematical theory, namely Minkowskian geometry. To apply Minkowskian geometry, we need to explain the notion of the *time of occurrence of an event.* This is necessary for defining a reference frame in which to measure motion. This notion is explained when we have a criterion for judging when two spatially separated events have the same time of occurrence; in this case, the criterion appeals to an equivalence relation between events (simultaneity). The explanation provided by Einstein’s criterion consists in its explication of when two events are simultaneous relative to an inertial frame by a procedure based on light signals, and so, in Minkowskian geometry, in terms of a construction based on paths that, according to the usual coordinating principles, are interpreted as the paths of light rays. The criterion, therefore, is the basis for the construction—the diagonal of the “light parallelogram”—that encodes the structure of Minkowskian geometry.^23^ In this way Minkowskian geometry is revealed to be the natural geometrical interpretation of a physical world with an invariant finite velocity. And note that because the criterion is empirically constrained, involving as it does nomological properties of emitted and reflected light signals, it not only controls the application of Minkowskian geometry but transforms it into a part of physics.

Einstein’s analysis of simultaneity is not, as the logical empiricists maintained, an “epistemological analysis” that merely supplies a coordinative definition or correspondence rule for giving an empirical meaning to a theoretical concept that had previously lacked one: it is an analysis, in several steps, of unacknowledged assumptions about the measurement of time and motion, the culmination of which is Einstein’s analysis of the problematic coordinative definition that is embedded in the Newtonian framework. This analysis of simultaneity and its place in an established conceptual framework reveals that the criterion for the concept’s application is tied to an empirical hypothesis, namely the hypothesis that the speed of light is the same in all reference frames. And with the provision of this criterion, we are led to a relation of simultaneity (relative simultaneity) that is fundamentally different from the relation that the Newtonian framework assumes (absolute simultaneity).^24^

### For motions

Let us return to the question of the equivalence principle’s methodological role. At the end of Sect. 2, we saw that the principle expresses the conditions under which we can judge that the motions of freely falling frames and Lorentz frames are identical. And, by this point, one might already think that the case for understanding the equivalence principle to express a criterion of identity is established. But there is another question that needs to be answered: can it be shown that the equivalence principle, so understood, controls the application of the mathematical framework of Einsteinian gravitation?

So far, we have discussed the equivalence principle only within the *context of theory development*. How might we formulate the principle in the *context of Einstein’s completed gravitation theory*? There are proposals by Trautman (1966, p. 321) and Anderson (1967, p. 335) that are particularly apt; here is a paraphrase: all non-gravitational experiments serve (approximately) to determine the same affine connection in a sufficiently local region of space–time.^25^ In more detail, what this formulation expresses is that, in a sufficiently local region of space–time such that tidal forces can be ignored, the laws governing non-gravitational interactions are constructed with the same affine connection. That is, no experiments reveal that certain physical processes couple to the gravitational field and not others, and so require a theoretical account involving a different affine connection. It is the universal coupling that this formulation captures that allows us to say that there exists in the neighbourhood of every point-event *p* a reference frame and an associated coordinate system such that the neighbourhood’s size and the first derivatives of the connection relate so that the connection components are close enough to constant, and so close enough to zero, and thus the laws governing non-gravitational interactions are the same as in special relativity.^26^

With the Trautman-Anderson formulation of the principle in hand, we can now formulate the following *criterion of identity for the motions of freely falling frames and Lorentz frames*: the motion of a freely falling frame is the same as the motion of a Lorentz frame if, and only if, all non-gravitational experiments determine the same affine connection in a freely falling frame as would be determined in a Lorentz frame.^27^ This formulation explicitly ties the equivalence principle to Lorentz frames and freely falling frames, to non-gravitational interactions and the laws governing them, to the geometric objects that figure in the laws, and in this way to the local affine structure of space–time.

Does this criterion of identity control the application of the theory of pseudo-Riemannian manifolds in Einsteinian gravitation? The criterion incorporates the affine connection, which figures in the geodesic equation^28^:

$$ \frac{{d^{2} x^{\mu } }}{{d\lambda ^{2} }} =  - \varGamma _{{\alpha \beta }}^{\mu } \frac{{dx^{\alpha } }}{{d\lambda }}\frac{{dx^{\beta } }}{{d\lambda }}. $$

When the affine parameter *λ* is the proper time along the path of a free particle, this is the assertion that “free massive test particles traverse time-like geodesics”; it is the counterpart, in Einsteinian gravitation, of the statement, in Newtonian theory, that “a body unacted upon by forces moves in uniform rectilinear motion.” The geodesic equation for light rays takes the same form, only proper time cannot be used as a parameter along the path of a light ray since the proper time interval between any points on it is zero. The corresponding principle for light rays is as follows: “light rays traverse light-like geodesics.” The geodesic equation gives an account of the behaviour of the most basic entities in Einsteinian gravitation, the motions of free particles and light rays. Hence, the criterion of identity, with its incorporation of the affine connection, picks out the geodesics with respect to the connection on which matter and light propagate. In this way—one that will be explained in greater detail in the next paragraph—the criterion controls the application of a pseudo-Riemannian geometry in Einsteinian gravitation. Note, furthermore, that because the criterion of identity is empirically constrained—it is based on the local indistinguishability of the motions of frames—the geometry is transformed into a part of physics.

It is worth elaborating on the criterion’s “controlling the application” of the space-time geometry of Einsteinian gravitation. To apply the space-time geometry, we need to explain the notion of the *inertial motion* or *inertiality* of a frame. For Einstein, this notion is explained when we have a criterion for judging that the motions of Lorentz frames and freely falling frames are the same motion—and in this case the criterion appeals to an equivalence relation between the motions of frames (local indistinguishability). The explanation provided by the criterion consists in its explication of when the motions of Lorentz frames and freely falling frames are locally indistinguishable, and so, in the space–time geometry of Einsteinian gravitation, when the non-gravitational interactions pick out the same affine connection and thereby the same local inertial structure. (Here the geodesics associated with the connection are interpreted, according to the usual coordinating principles, as the paths of free particles and light rays.)

There are two objections to the foregoing that I wish to address—they both serve to clarify my account. One might object that the criteria of identity for times and motions are different from those for numbers and lengths. The identity is established at a second level in the cases of times and motions: “these concepts have the same number of objects falling under them” and “these tracts are the same length” are established by a single direct comparison, whereas “these events occur at the same time” and “the motions of these frames are the same” are arrived at on the basis of the systematic agreement of several measurements or kinds of measurements. But the fact that the identity at issue in the latter two cases is a higher-order property is not an objection but a clarification. What matters is that in all of these cases—however the identity is established and at whatever level—the criteria of identity express the conditions under which we can judge that the same thing has been presented to us in two different ways.

One might also object that the criterion of identity for motions is different from the other criteria of identity considered. Criteria of identity tell us when two objects are of the same kind and, in the first three cases, like objects are compared, but not in the last. In more detail, the objection runs as follows: the criterion of identity for numbers identifies when two sets are of the same kind, i.e., when they are equinumerous; the criterion of identity for lengths identifies when two tracts are of the same kind, i.e., when they are congruent; the criterion of identity for times identifies when the times of two events are of the same kind, i.e., when they are simultaneous. But the criterion of identity for motions does not compare like and like: the motions of Lorentz frames and the motions of freely falling frames. Now, the criterion may collapse the distinction and show that these motions are of the same kind in the new theoretical framework that Einstein is defending, but that still distinguishes it from the others. Let me make two observations. First, the objection appeals to our pre-analytic conceptions of numbers, lengths, and times, and to the idea that they can be compared unproblematically; motions are set apart. This reflects a lack of caution about our presuppositions regarding what is demanded for, e.g., an event to have a time of occurrence. This neglects that the explications of number, length, time, and inertia are informed by their respective criteria of identity. Second, it is assumed that the role of a criterion of identity is to establish when two objects are of the same kind (concept). But Frege is very clear that on his use of the notion, the criterion of identity tells us when the same object has been presented to us in two different ways. For Frege, criteria of identity deal specifically with objects, not kinds (concepts). The analysis of the concepts at issue—number, length, time, inertial motion—is informed by the criteria of identity, but the criteria of identity themselves deal with objects not concepts. In the same way as the other criteria of identity, therefore, the criterion of identity for motions tells us when the same thing, the inertial motion or inertiality of a frame, has been presented to us in two different ways.^29^

In overview, the interest of the criteria of identity that underlie Frege’s and Einstein’s analyses resides in the *equivalence relations* they express. As Demopoulos has stressed, both Frege and Einstein take the equivalence relations—found on the right-hand side of the criteria of identity—as primary and then proceed to explain the relevant recognition judgements—found on the left-hand side—in terms of the holding of the appropriate relation. In each analysis, the criterion of identity emerges from the attempt to reveal the presuppositions on which the use, misuse, and limitations of the concepts at issue—number, length, time, and inertial frame—depend. Frege shows that the analysis of number is informed by a criterion of identity for numbers and that this criterion is not empirically constrained. By contrast, Einstein’s analyses of length, time, and inertial motion turn on criteria of identity that are founded on a host of empirical hypotheses; in this way, they are empirically constrained and the applied mathematical theories they control are transformed into a part of physics. But in spite of the difference between criteria of identity that are and are not empirically constrained, it should be clear that none of those just considered—for numbers, for lengths, for times, for motions—are mere stipulations or conventions. And while the latter three are empirically constrained, they are not founded on simple induction either, but reflect an interplay of mathematical and interpretive considerations: they enable us to define and interpret concepts such as length, time, simultaneity, and inertia—and so all those that are related to them.

Each criterion of identity is an answer to the question, by virtue of what *principle* are objects *x* and *y* identified? In each case, the claim that the principle in question is a criterion of identity is not intended as a novel interpretive claim about a significant proposition in the exact sciences, but as a natural reconstruction, from a standpoint of greater conceptual clarity, of what underlies Frege’s and Einstein’s analyses. In each case, it is argued, the analysis turns on the provision of a criterion of identity. The foregoing discussion is summarized in the following table:

**Table:** 

	Concept analyzed	Criterion of identity	Equivalence relation	Criterion of identity empirically constrained	Mathematical theory applied
Frege	Number	For numbers	Equinumerosity	No	Theory of the natural numbers
Einstein	Length	For lengths	Congruence	Yes	Geometries of constant curvature
Einstein	Time	For times	Simultaneity	Yes	Minkowskian geometry
Einstein	Inertial motion	For motions	Local indistinguishability	Yes	Theory of pseudo-Riemannian manifolds

## Significance

I have argued that Einstein’s 1907 analysis of the Lorentz frame turns on his provision of a criterion for recognizing the motions of freely falling frames and Lorentz frames as the same motion. This new account illuminates the methodological role of the equivalence principle; it also clarifies the account of the application of a pseudo-Riemannian geometry in Einsteinian gravitation. But there is a further implication: this account isolates what is distinctive about Einstein’s contribution to our understanding of the gravitational interaction. Isolating what is distinctive is important because it is sometimes claimed that the equivalence principle was already there in the *Principia*—in Corollary VI to the Laws of Motion. For example, Saunders (1998, p. 148, 2013, p. 37) has claimed that Corollary VI is the equivalence principle; Knox expresses essentially the same view (2014, p. 11).

It is worth recalling the context in which Corollary VI appears and what it is used to establish. Corollary VI is part of the argument running the length of the *Principia* for a solution to the “Two Chief World Systems” problem. It is an argument that begins with the laws of motion, the corollaries to the laws, and all of the propositions proved in Book 1 of *Principia*; that considers the extension of this framework to the celestial realm; that proceeds to an estimation of the masses of the Solar System bodies and the calculation of the system’s centre of mass; and whose conclusion is both a solution and a transformation of the original problem. What follows is only a schematic account of the role of Corollary VI in this argument.

The laws of motion express criteria for the application of the conceptual framework of Newtonian mechanics. With this framework in hand and with the assurance that it applies to cases of mechanical interaction (impact), Newton asks whether it can be extended to the long-range centripetal forces holding the planets and satellites in their orbits. He postulates that the third law of motion holds between all bodies in the universe. Now supposing that it does hold and that every body attracts every other reciprocally according to some yet-to-be-determined force law, Newton asks the following question: how, using the framework of the laws of motion, can we give an account of the motions within a particular subsystem of bodies when the system is acted upon by every other body? How, given the complexity of these interactions, is the analysis of orbital motion possible? This would seem to pose an intractable problem.

But Newton uses Corollary VI to show that certain subsystems, namely planetary systems such as that of Jupiter and its satellites, that are far enough away from large gravitating bodies suffer a gravitational attraction, but the lines of force can be treated as very nearly equal and parallel and the systems behave as though they suffered no gravitational attraction at all. Newton expresses this in Corollary VI as follows:

If bodies are moving in any way whatsoever with respect to one another and are urged by equal accelerative forces along parallel lines, they will all continue to move with respect to one another in the same way as they would if they were not acted on by those forces.

The corollary implies that the influence of distant bodies on certain subsystems can be ignored, that is, these systems can be treated as effectively isolated. Newton appeals to the corollary in Book 1, Proposition 66, Case 1 and in Corollary 19 of the same proposition.^30^

Corollary VI establishes that bodies in centre-of-mass systems that are freely falling towards distant stars behave among themselves just as they would if the system were moving inertially. That is to say, the outcomes of all mechanical experiments in a freely falling centre-of-mass system are the same as would be obtained in an inertial frame.

Does Corollary VI have the same empirical content as the equivalence principle? Newton “derives” Corollary VI from the laws of motion and it is reasonable to think that he expects it to apply to all interactions.^31^ After all, Corollary VI says nothing about what kinds of forces urge the bodies along lines that are very nearly equal and parallel. What is questionable is whether he was prepared to make a blanket, exceptionless statement such as the equivalence principle. For example, even if Newton assumed that every interaction obeys the third law, he might not have been confident that light, which he took to consist of massive corpuscles, must also fall like massive particles. What seems clear is that Corollary VI contains no claim so explicit as the principle of universal coupling, and therefore cannot be said to have the same empirical content as the equivalence principle.^32^

Does Corollary VI function as a criterion of identity for the motions of inertial frames and freely falling frames? Corollary VI establishes that these motions, at least in the Newtonian framework, cannot be distinguished by experiment, but it does not undermine the theoretical distinction: inertial motion and freely falling motion are distinct in the *Principia*. That is to say, *Corollary VI does not function as a criterion of identity*. Newton is concerned to show that a “Corollary VI frame” can be treated as a near-enough approximation to an inertial frame.

It is important to understand why the distinction between inertial and non-inertial motions, inertial and non-inertial frames, is maintained. In the *Principia* and at the end of the *Opticks* Newton outlines a program of empirical investigation on the basis of his dynamical theory. Both the program and the dynamical theory are based on the notion of a “force of nature,” and so on the laws of motion as a (partial) explication of that notion. The dynamics does not require the notion of absolute velocity, nor that of absolute space, but it does require (“absolute”) acceleration—and *this* presupposes a distinction between inertial and non-inertial motions. Without this distinction, the Newtonian conception of a moving force cannot be articulated. Therefore, in spite of Corollary VI, it is difficult to see how the dynamical theory can be weakened without changing it essentially.

Now, going beyond the conceptual framework of *Principia*, we can of course identify freely falling frames and inertial frames; this is what is done in Newton-Cartan theory. The identification is integral to Cartan’s reconstruction of Newtonian gravitation, a reconstruction that is in many respects natural and instructive, but it is an identification that Newton could not countenance and that is alien to the structure implicit in *Principia*.

I have argued, in sum, that a careful study of the equivalence principle clarifies two things: Corollary VI has *neither* the same empirical content as the equivalence principle *nor* the same role. In this way this new account of the principle isolates what is distinctive about its contribution to gravitation theory, and so it benefits our understanding of the foundations of both Einstein’s and Newton’s theories.

There are two further implications of this account that I wish to draw out. First, this account substantiates the idea that the notion of a criterion of identity has something to offer the analysis of physics. The criterion of identity, though originating in the foundations of mathematics and taken up by philosophers working in general metaphysics, has not previously been employed in the service of the foundations of physics. It is worth noting that this employment of the criterion of identity is part of a methodological—and therefore epistemological—analysis and not a metaphysical project. It has nothing to do with individuation, objects, kinds or sortals in the usual senses of these terms; still less anything to do with identifying “surplus structure” or playing an otherwise eliminative role. The criterion of identity, while it does identify two previously distinct motions, has a “synthetic” or constructive role: it motivates a reinterpretation of free fall and with it a new framework of empirical investigation, one in which the distinction between inertial and non-inertial frames is replaced by a new distinction between freely-falling and non-freely-falling frames.

Second, the account is also of more general importance to the history and philosophy of science. It offers an alternative to two prominent accounts of theory change, namely Kuhn’s (1962) and the conventionalists’ (e.g., Reichenbach 1928 [1958]; Carnap 1934 [1951]; Grünbaum 1963). Kuhn held defenders of different paradigms to be inhabitants of different worlds who cannot argue with one another because there are no paradigm-transcendent criteria of rationality that could make such argument possible. The transition from one paradigm to another therefore is the result of an extra-rational process. For the conventionalists, the transition from one theoretical framework to a new one is a matter of expediency. These accounts have no better means of explaining the transition from the special-relativistic framework to a new framework than by way of the problematic notions of a Kuhnian paradigm shift and a change of conventions. In neither account is there any appreciation of the considerations in the context of theory development that motivate the transition.

In contrast with Kuhn and the conventionalists, I have shown that in the case of Einsteinian gravitation this transition was the result of a conceptual analysis. The key step in this analysis was showing that the Lorentz frame is not uniquely determined by its empirical criteria, and it was Einstein’s recognition of a particular *equivalence relation*—what is expressed in the equivalence principle—that establishes this. I have argued that the principle functions as a criterion of identity and that the principle has a substantive and manifestly constructive role: it motivates a new concept of natural motion. To regard the principle, then, as a mere heuristic at best diminishes, at worst obscures, its role. This account, though a development of Demopoulos’ novel employment of the notion of a criterion of identity, is not intended as a radical interpretation of the principle, but as an altogether natural way of understanding its methodological role in the conceptual framework of gravitation theory. In several respects it is surprising that it has not already been proposed.

## Footnotes

1 Einstein’s first formulation of the principle can be found in his article “On the Relativity Principle and the Conclusions Drawn from It” (1907, p. 454). Other early accounts include his “On the Influence of Gravitation on the Propagation of Light” (1911, pp. 898–899), his review article “The Foundation of the General Theory of Relativity” (1916a, pp. 772–773), his note “On Friedrich Kottler’s Paper: ‘On Einstein’s Equivalence Hypothesis and Gravitation’” (1916b, p. 639), his popular exposition *Relativity: The Special and the General Theory* (1916c [2004], Chapter 20), and his Princeton Lectures (1922, Lecture III). So far as I know, the first time he uses the term “principle of equivalence” is in his reply to Kottler. Further remarks about Einstein’s insight of 1907, including his remark that it was “the happiest thought of my life,” can be found in his “Fundamentals and Methods of the Theory of Relativity” (1920). It is worth noting, however, that the principle would find a more precise formulation only much later in the general relativity literature.

2 It is worth noting that all three laws of motion are necessary to give an account of inertial motion—the first law is sufficient only for ideal point-particles. This is clear in Newton’s account of inertia. Hence, referring to the first law as “the principle of inertia” encourages the widespread view that it alone is enough. So far as I know, Newton himself did not use the term. I do not know at what point it appeared, but it can be found in Euler’s “Réflexions sur l’espace et le temps” (1748), if not in any earlier source. See (Samaroo, 2017) for a detailed account of the role of each of the three laws in the account of inertia.

3 Other notable contributors include Mach (1883 [1919]), Muirhead (1887) and MacGregor (1893).

4 It would be shown later in the twentieth century that the laws governing all non-gravitational interactions are Lorentz invariant.

5 There have been many taxonomies of equivalence principles, but it is worth noting the influential one due to Clifford Will (1993). Will distinguished between the weak, Einstein, and strong equivalence principles. He characterized the weak principle in the same way as I have. And it is worth noting that the weak principle was tested by laboratory experiments up to Einstein’s time, whereas Einstein’s hypothetical extension, which we will come to shortly, requires new tests. In Will’s taxonomy, the Einstein equivalence principle is the claim that “(i) WEP is valid; (ii) the outcome of any local nongravitational test experiment is independent of the velocity of the (freely falling) apparatus; (iii) the outcome of any local nongravitational test experiment is independent of where and when in the universe it is performed.” (1993, Chapter 2) Will’s strong principle modifies (i) by specifying that it holds not only for test matter but also for self-gravitating bodies (1993, Chapter 3). This characterization is unorthodox and, further on, I will contrast it with that of Anderson (1967) and Ehlers (1973).

6 This is easy to show. Consider the law of universal gravitation $\left| {\varvec{F}_{12} } \right| = \, (G\mu_{1} \mu_{2} )/\left| {\varvec{x}_{1} {-}\varvec{x}_{2} } \right|^{2}$, where ***F***_12_ is the force on particle 1 of gravitational mass μ_1_ due to particle 2 of gravitational mass μ_2_. The equality or proportionality of inertial mass and gravitational mass implies that μ_i_ in this equation may be replaced by *m*_i_ (from the second law of motion) to obtain $\left| {\varvec{F}_{12} } \right| = \, (Gm_{1} m_{2} )/\left| {\varvec{x}_{1} {-}\varvec{x}_{2} } \right|^{2}$. The acceleration ***a***_1_ of particle 1 due to this force is given by ***F***_12_ = *m*_1_***a***_1_. Hence, $m_{1} \varvec{a}_{1} = \, (Gm_{1} m_{2} )/\left| {\varvec{x}_{1} {-}\varvec{x}_{2} } \right|^{2}$. Both sides can be divided by *m*_1_, so the acceleration of any body subject only to gravitation will be independent of its mass.

7 This can be found in *The Evolution of Physics* (Einstein and Infeld 1938, pp. 226–235).

8 For a good discussion of tidal forces, see Ohanian and Ruffini (1994).

9 Note that this principle differs from the one Will (1993) calls “the Einstein equivalence principle”—we will come to that principle further on. For further details on Einstein’s understanding of the equivalence principle, see Norton (1985). But note that, although Einstein did attach particular importance to the gravity-producing version of the principle, on at least one occasion he expressed it as a transforming-away principle regarding reference frames. See his Princeton Lectures (1922, pp. 67–68).

10 This is also readily illustrated by Einstein’s elevator. Consider the gravity-producing version. Suppose you stand in the elevator cabin. You feel a “gravitational force” towards the floor, just as you would at home. And, as before, there is no way of excluding the possibility that the cabin is part of an accelerating rocket in free space, and that the force you feel is an accelerative force. But this time there is a light source affixed to one of the cabin walls that emits a beam horizontally. Photons carry energy, so even though they have no mass, they have momentum and the beam emitted will not travel across the cabin horizontally to hit a point opposite its point of emission, but will curve downwards towards the floor—in analogy with a ball thrown horizontally in the gravitational field of the Earth. Assuming that the slight curve of its path were measurable, the beam cannot distinguish the cabin on Earth from the cabin that is part of the accelerating rocket.

11 Given this bold hypothesis, it is significant that the first test of Einsteinian gravitation was the observation of the deflection of starlight in the eclipse observations of 1919. This was an important step in vindicating universal coupling.

12 In the taxonomy of Will (1993) and other standard references, this principle (and close relatives with the same essential content) is known as “the Einstein equivalence principle”; it can be found in Einstein’s Princeton Lectures (1922, pp. 67–68), so the name is clearly not misapplied. Evidently, it is different from the principle that I, following Norton (1985), have called “Einstein’s own equivalence principle” in that it incorporates universal coupling.

13 By “sufficiently local,” what is meant is that the region is small enough for gravitational forces to act nearly equally and along parallel lines.

14 The argument, implicit in the foregoing, is the following: (P1) If the Lorentz frame is uniquely determined by its empirical criteria, then an observer floating in an elevator cabin should be able to tell whether he is in free space or freely falling in a gravitational field. (P2) An observer in an elevator cabin cannot tell whether he is in free space or freely falling in a gravitational field. (C) Therefore, the Lorentz frame is not uniquely determined by its empirical criteria.

15 Recall that in Newtonian theory the proportionality of inertial mass *m* to gravitational mass *µ* is a remarkable fact that lacks an explanation. This proportionality is explained by way of the equivalence principle. The principle establishes that *immersion in a homogeneous gravitational field and uniform acceleration are identical in their effects*. Since the two concepts of mass figure in the expressions for gravitational force and accelerative force, the principle implies that *inertial and gravitational mass are not merely proportional or equivalent but “identical in essence.”* Einstein himself (1912, p. 1063, 1918, p. 241) used the suggestive term *wesensgleich*—identical in essence, essentially the same, consubstantial—to express this identity.

16 See Pitts (2016a, b, 2018) for details and for a near-exhaustive list of the original research papers.

17 For example, in massive spin-2 gravity, immersion in a homogeneous gravitational field and uniform acceleration are not identical in their effects. The difference between gravitational effects and inertial effects is observable only in experiments sensitive to the graviton mass term in the gravitational field equation, that is, only if one looks carefully enough to observe the influence of the mass term on inertial effects. See Pitts (2016a, b, 2018) for details.

18 Frege claimed to have reduced arithmetic to logic—the familiar construal of logicism—even though he understood this reduction in a very particular way. He defends this claim by offering arguments that aim to establish the analyticity and apriority of arithmetic. In contrast with Frege’s own defence, Demopoulos (2013, Chapter 1) has argued for a reconstruction of *Grundlagen* that is independent of the truth of logicism or the doctrine of logical objects, and of Frege’s views about primitive truths and their natural order.

19 Demopoulos’ claim that the notion of a criterion of identity is relevant to the analysis of space–time theories can be found in Chapter 2 of *Logicism and its Philosophical Legacy* (2013). His first example is that of Euclidean geometry, which, together with the principle of free mobility (understood as a criterion of identity for lengths), becomes a space–time theory. His second example is that of Minkowskian geometry, which, together with the Einstein synchronization criterion (understood as a criterion of identity for times), becomes a space–time theory.

20 In fact, the concepts of practically rigid body and congruence are interdefinable and the principle of free mobility expresses a criterion for the application of both at once.

21 As is well known, the principle of free mobility controls not only the application of Euclidean geometry but also that of elliptic geometry and hyperbolic geometry, that is, the geometries of constant positive and negative curvature. The geometrical result that underlies this is the Helmholtz-Lie theorem. See Stein (1977) and Torretti (1978) for details.

22 Demopoulos’ claim that the notion of a criterion of identity can be employed more widely than in the foundations of arithmetic is foreshowed in *Grundlagen*. Frege (1884 [1989], §64–67) claims that the notion of the direction of a line turns on the provision of a *criterion of identity for directions*: the direction of *l* is the same as the direction of *m* if, and only if, *l* is parallel to *m*. See Demopoulos (2013, pp. 187–188) on the role of this discussion in Frege’s account of the criterion of identity.

23 Demopoulos’ (2013, pp. 37–38) account tacitly presupposes that the world-lines of free particles define a projective structure, and that projective structure plus the conformal structure defined by the propagation of light determines the metric (up to a scale factor). While Demopoulos does not reference Weyl (1918, 1921, 1921) and Ehlers et al. (1972), he takes for granted their results.

24 See DiSalle (2006, Chapter 4; 2010, section 2) for a careful examination of Einstein’s analysis.

25 The affine connection is the geometric object that, in Newtonian terms, expresses the gravitational field strength; in Einsteinian terms, it expresses how an observer in motion relative to you moves so that, from his point of view, you are travelling in a straight line.

26 Now, in addition to the equivalence principle, there is another principle that is necessary to ensure that special relativity is valid as a local approximation—the equivalence principle alone does not do this. The principle in question is the *principle of minimal coupling*, according to which no terms of the special-relativistic equations of motion contain gravitational variables. (For a more careful account of the principle of minimal coupling, see Brown and Read (2016)). The equivalence principle, together with the principle of minimal coupling, is known as the *strong equivalence principle*. (So far, my characterizations of the “weak” and “Einstein” principles have accorded with the taxonomy of Will (1993). This characterization of the strong principle follows Anderson (1967) and Ehlers (1973); it differs from Will’s, which does not assume minimal coupling and which differs from the Einstein principle in its inclusion of bodies with gravitational self-energy). It is noteworthy that Anderson (1967) claims that only the equivalence principle is integral to Einsteinian gravitation, not the strong principle. By contrast, Brown (2005) leaves it as an open question. The question about the status of minimal coupling is an important one. But my account does not require me to pronounce on this either way.

27 Evidently, this criterion of identity, which incorporates the Trautman-Anderson version of the equivalence principle, represents an ideal, with something only approximate actually holding. For one thing, it is assumed, in the case of the freely falling frame, that the size of the experiment is sufficiently small in relation to the distance scales picked out by the Riemann curvature tensor; in this way, tidal forces can be ignored.

28 Note that Weinberg (1972, Chapter 3, section 2) offers a now-familiar derivation of the geodesic equation of motion from the equivalence principle.

29 In a similar vein to this last objection, one might say that the criteria of identity for numbers, lengths, and times are more straightforward than the criterion of identity for motions. One might argue that “the motions of these frames are the same” rests on more physical assumptions than, e.g., “these events occur at the same time.” For example, one might argue that a frame falling in a weak gravitational field, far removed from the nearest large mass, has the same motion as a Lorentz frame, but not the frame of a particle falling into a black hole. But in each of the three cases where the criteria of identity are empirically constrained, various assumptions must be made: that the tracts are (practically) rigid in the case of the criterion of identity for lengths; that space is isotropic in that of the criterion of identity for times; that strong gravitational fields are excluded in that of the criterion of identity for motions.

30 But it is worth noting that the role of Corollary VI in Newton’s argument is far more explicit in *The System of the World*, notably in articles 8 and 27.

31 It is central to Newton’s argument that gravitation is proportional to mass, without regard to the kind of matter or, e.g., charge. This is evident in his discussion of his pendulum experiments in Book 3, Proposition 6 of the *Principia*.

32 One might say that, within the Newtonian framework, it is yet-unknown (regardless of what Newton expected) whether the outcomes of all non-gravitational experiments will be the same in freely falling frames as would be obtained in inertial frames, and so will determine the same affine connection in a given region of space–time.
